# 658. Diagnostic Testing Among Patients with Suspected Recurrent *Clostridioides difficile* Infection (rCDI) in ECOSPOR III a Phase 3 Clinical Trial: Implications for Clinical Practice vs Clinical Trials

**DOI:** 10.1093/ofid/ofab466.855

**Published:** 2021-12-04

**Authors:** Matthew Sims, Sahil Khanna, Darrell Pardi, Paul Feuerstadt, Charles Berenson, Henry Wu, Elaine E Wang, Elaine E Wang, Barbara McGovern, Lisa von Moltke

**Affiliations:** 1 Beaumont Hospital, Royal Oak, MI; 2 Mayo Clinic, Rochester, MN; 3 Yale University School of Medicine/PACT-Gastroenterology Center, Westport, CT; 4 State University of New York at Buffalo, Buffalo, NY; 5 CR Medicon Research, Piscataway, New Jersey; 6 Seres Therapeutics, Cambridge, MA

## Abstract

**Background:**

Accurate diagnosis of rCDI is challenging because of limitations in test performance and alternative causes of recurrent diarrhea, such as post-infectious irritable bowel syndrome (IBS). Stool enzyme immunoassay (EIA) toxin testing (TOX) is the best predictor of active disease, but may miss cases of CDI when toxins are below the limit of detection. In contrast, glutamate dehydrogenase (GDH) or PCR have high sensitivity but cannot differentiate colonization from infection, leading to possible overdiagnosis due to low specificity. In ECOSPOR III, SER-109, an investigational purified microbiome therapeutic, was superior to placebo in reducing rCDI (12.4% vs 39.8%, respectively; p-value < 0.001). We examined diagnostic testing patterns among screened subjects.

**Methods:**

Patients with ≥2 prior episodes and ≥3 unformed bowel movements over 48 hours were screened. To ensure enrollment of patients with active CDI, toxin testing was required at entry via a local certified or central lab (Eurofins; Framingham, MA). Subjects with discordant GDH+/TOX- tests at the central lab had reflex confirmatory testing with a cell cytotoxicity neutralization assay (CCNA), considered the “gold standard” for toxin testing.

**Results:**

The leading reason for screen failure among 281 subjects screened was a negative toxin test (50/99; 50.5%). Of 182 patients enrolled, 59 (32.4%) qualified with EIA TOX+ at the local lab (33 TOX+; 25 GDH+/TOX+) and 122 (67.0%) qualified by the central lab (Table 1). Of these 122 subjects, 87 qualified by GDH+/TOX+ but 35 required additional reflex testing by CCNA due to discordant GDH+/TOX-results; all 35 were positive.

Diagnostic Testing for Qualifying C. difficile Episode in ITT Population

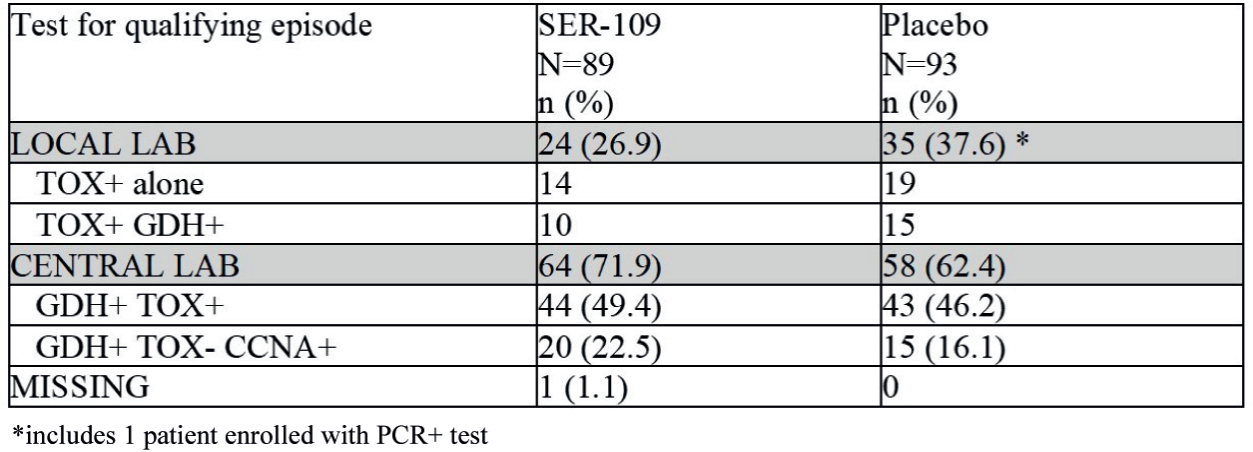

**Conclusion:**

These diagnostic testing patterns suggest a subset of patients with suspected rCDI have toxin concentrations below the EIA threshold for detection or may have an alternative cause of diarrhea, such as post-infectious IBS. Thus, the limitations of EIA toxin testing need to be considered in clinical practice when evaluating patients with compatible symptoms of rCDI and a high prior probability of infection. In contrast, in trials of investigational agents, toxin testing assures enrollment of patients with active disease and accurate estimates of efficacy.

**Disclosures:**

**Matthew Sims, MD, PhD, FACP, FIDSA**, **Astra Zeneca** (Independent Contractor)**Diasorin Molecular** (Independent Contractor)**Epigenomics Inc** (Independent Contractor)**Finch** (Independent Contractor)**Genentech** (Independent Contractor)**Janssen Pharmaceuticals NV** (Independent Contractor)**Kinevant Sciences gmBH** (Independent Contractor)**Leonard-Meron Biosciences** (Independent Contractor)**Merck and Co** (Independent Contractor)**OpGen** (Independent Contractor)**Prenosis** (Independent Contractor)**Regeneron Pharmaceuticals Inc** (Independent Contractor)**Seres Therapeutics Inc** (Independent Contractor)**Shire** (Independent Contractor)**Summit Therapeutics** (Independent Contractor) **Sahil Khanna, MBBS, MS**, **Seres** (Grant/Research Support) **Darrell Pardi, MD**, **seres** (Consultant)**Vedanta** (Consultant) **Paul Feuerstadt, MD, FACG**, **Ferring/Rebiotix Pharmaceuticals** (Consultant, Scientific Research Study Investigator, Speaker's Bureau)**Finch Pharmaceuticals** (Scientific Research Study Investigator)**Merck and Co** (Speaker's Bureau)**SERES Therapeutics** (Consultant, Scientific Research Study Investigator)**Takeda Pharmaceuticals** (Consultant) **Elaine E. Wang, MD**, **Seres Therapeutics** (Employee) **Elaine E. Wang, MD**, **Seres Therapeutics** (Employee, Shareholder) **Barbara McGovern, MD**, **Seres Therapeutics** (Employee, Shareholder) **Lisa von Moltke, MD**, **Seres Therapeutics** (Employee, Shareholder)

